# Biosynthesis of Silver Nanoparticles Using *Tagetes erecta*: Extract Characterization, Morphological Modification Using Structure Directing or Heterogeneous Nucleating Agents, and Antibacterial Evaluation

**DOI:** 10.3390/molecules30234596

**Published:** 2025-11-29

**Authors:** Edgar J. López-Naranjo, Margarita Cid-Hernández, Milton O. Vázquez-Lepe, Marisol Luviano, María Judith Sánchez-Peña, Luis J. González-Ortiz, César A. Dueñas-Bolaños, Jaime A. Jiménez-Aguilar, Luisa Fernanda Briones-Márquez, Azucena Herrera-González

**Affiliations:** CUCEI-Centro Universitario de Ciencias Exactas e Ingenierías, Universidad de Guadalajara, Guadalajara C.P. 44430, JAL, Mexico; margarita.cid@academicos.udg.mx (M.C.-H.); milton.vazquez@academicos.udg.mx (M.O.V.-L.); marisol.luviano1389@alumnos.udg.mx (M.L.); maria.spena@academicos.udg.mx (M.J.S.-P.); luisj.gonzalezo@academicos.udg.mx (L.J.G.-O.); cesar.duenas7449@alumnos.udg.mx (C.A.D.-B.); jaime.jimenez9036@alumnos.udg.mx (J.A.J.-A.); fernanda.briones@academicos.udg.mx (L.F.B.-M.); mariaa.herrera@academicos.udg.mx (A.H.-G.)

**Keywords:** biosynthesis, nanoparticles, non-toxic agents, morphological modification, antibacterial activity

## Abstract

This work reports the biosynthesis of silver nanoparticles (AgNPs) using an autoclave method with *Tagetes erecta* extract (TEE) as a source of reducing agents, silver nitrate (AgNO_3_) as the metal precursor, and a nucleating agent (i.e., sodium chloride [S]) or a structure director agent (i.e., gum Arabic [G] or hydrous magnesium silicate/talc powder [T]) to tailor the morphology of AgNPs. Since the properties and potential applications of AgNPs depend on their size and shape, these additives were employed to achieve morphological control. Phytochemical screening tests and UPCL-Qtof-MS/MS profiling of TEE were performed to identify the compounds present in the extract, indicating that highly polar phenolic compounds such as saponins, tannins, and flavonoids are present in TEE, allowing it to act as a source of reducing/stabilizing agents. The biosynthesized AgNPs exhibited different morphologies (i.e., spheres, rods, ribbons, and wires) depending on the modifying agent used (i.e., S, G, or T). Characterization techniques including scanning electron microscopy (SEM), transmission electron microscopy (TEM), ultraviolet–visible spectroscopy (UV–vis), and X-ray diffraction (XRD) confirmed the successful use of S, G, and T in modulating AgNP morphology. The results of the antibacterial activity evaluation demonstrated that both TEE and AgNPs possess bacteriostatic activity against *Escherichia coli* and *Enterococcus faecalis*, with the use of S as a nucleating agent increasing the inhibitory effect of AgNPs.

## 1. Introduction

The morphology of silver nanoparticles (AgNPs) is of particular significance since it determines their properties and applications. In addition to typical morphologies such as spheres, wires, and rods, uncommon shapes like polygons, flower-like particles, and pyramids have demonstrated to be effective as antibacterial agents or plasmonic active structures in optical sensing and imaging due to their large effective surface. Through the control of reaction parameters like temperature, reactant ratio stirring rate, or the use of additional reagents such as a structure director or nucleating agents, it is possible to modify the morphology of the synthesized nanostructures. AgNPs can be obtained by means of different synthesis routes, which are classified into three main groups: chemical, physical, and biological. Chemical methods are the most widely used; however, they employ organic solvents and toxic compounds that show hazardous effects on the environment and human health. On the other hand, physical routes show low productivity and consume a large amount of energy, which raises production costs. Finally, biological methods, particularly plant extract-based routes, have attracted worldwide attention due to their inexpensive, environmentally friendly, fast, and simple process. Biomolecules such as polysaccharides, tannins, phenolics, saponins, and terpenoids reduce and stabilize silver ions to obtain AgNPs [1,2,3,4,5,6,7,8]. *Tagetes erecta*, a plant native to Mexico, is widely used in garlands for social and religious purposes; its flowers and leaves have been previously employed in the synthesis of AgNPs with silver nitrate (AgNO_3_) as a metal precursor, obtaining spherical and/or spherical-like nanoparticles. In order to increase the possible applications of *Tagetes erecta*-AgNPs or any other biosynthesized nanoparticles, a wide variety of morphologies is needed [2,9,10]. It is known that nucleating agents induce the formation of seeds for the heterogeneous nucleation of metallic Ag, while structure-directing agents modify the size and shape of AgNPs through a preferential binding of the agent on specific crystal facets [11]. In this regard, Samanta et al. [12] reported the chemical synthesis of AgNPs of different morphologies by a seeding growth approach using seeds prepared by reducing silver nitrate (AgNO_3_). Moreover, Schuette et al. [13] used sodium chloride (S) as a nucleating agent for the growth of silver nanowires, employing a chemical route. AgNPs have shown antimicrobial properties against a wide variety of pathogenic microorganisms, including multidrug-resistant bacteria, which are related to their various mechanisms of action that attack microorganisms in multiple structures at a time. The effectiveness of AgNPs against microorganisms can be modified by their chemical composition [14,15,16]. Thus, in the present work, an attempt has been made to modify the morphology of *Tagetes erecta*-AgNPs by employing a heterogeneous nucleating agent (i.e., S) and structure-director agents (i.e., gum Arabic [G] or talc powder [T]) and to evaluate the effect of such additional agents on the antibacterial activity of the biosynthesized AgNPs against *Enterococcus faecalis* and *Escherichia coli*.

## 2. Results and Discussion

### 2.1. Extract Characterization

#### 2.1.1. Phytochemical Screening of TEE

Results obtained from the screening tests (Table 1) indicate the presence of glucoside saponins and flavonoids. Additionally, an average concentration of 0.1440 mM of Fe^2+^ was determined before starting the synthesis. However, at the end of the silver reduction (Ag^1+^), 0.0278 mM of Fe^2+^ was available in the medium, indicating a total consumption of 0.1162 mM of Fe^2+^. On the other hand, regarding the consumption of available reducing sugars, an average equivalent to 0.0160 mM of reduced sugars was quantified.

#### 2.1.2. TEE UPLC-Qtof-MS/MS Profile

Table 2 shows that the UPLC-Qtof-MS/MS profile of the aqueous *Tagetes erecta* flower extract is dominated by highly polar phenolics, with flavonol O-glycosides and O-acylglycosides as the central motif, accompanied by early-eluting organic and phenolic acids. Under the present conditions (formic acid in both mobile phases), intact glycosides frequently appear as formate adducts [M+HCOO]^−^, while deprotonated molecules [M−H]^−^ prevail for aglycones and small acids. This ionization balance produces characteristic adduct deprotonated pairs separated by +46 u that are visible throughout the chromatographic window and greatly aids class-level assignment. At the head of the gradient, quinic acid ([M−H]^−^ *m*/*z* 191; fragments 173/127/111) and gallic acid ([M−H]^−^ *m*/*z* 169 → 125, CO_2_ loss) anchor the hydrophilic end of the profile, consistent with an aqueous extraction enriched in water-soluble phenolics. The core region (≈3.5–7.0 min) is built from a series of flavonol conjugates that display the expected neutral losses of sugar and acyl groups (−162 u (hexose), −86 u (malonyl), and −179/161 u (caffeoyl)) and resolve to aglycone anchors at *m*/*z* 317 (quercetagetin), 331 (patuletin), 301 (quercetin), and 285 (kaempferol). Within this landscape, multiple isorhamnetin-3-O-glucuronide isomers elute between ≈6.07 and 6.50 min, each showing [M−H]^−^ ≈ 491.24–491.28 and occasional [2M−H]^−^ dimers around *m*/*z* 983; their co-elution with acylated flavonols and the appearance of adducts near *m*/*z* ~591 are typical in formate-rich media and corroborate glucuronidation of flavonols in polar matrices. Higher-mass features further reinforce this pattern. A strong adduct at *m*/*z* 864 is consistent with a tri-glycosylated laricitrin species, whereas an adduct at *m*/*z* 787 accompanied by the diagnostic ladder 575 → 463 → 331 supports a laricitrin dihexoside bearing malonyl substitution. These oligoglycosides are chemically reasonable outcomes of an aqueous extraction, where multiple sugar units increase solubility and retention falls in the late–mid window. Importantly, the feature at RT 2.07 min is conclusively assigned to ellagic acid, [M−H]^−^ theoretical *m*/*z* 300.9984, observed ~301.000 (−5.2 ppm), with canonical fragments at *m*/*z* 257 and 229. Although the ellagic/ellagitannin contribution is modest relative to the flavonol load, its presence is analytically valuable and aligns with phenolic surveys in related plant matrices. Mass-accuracy behavior follows expectations; [M−H]^−^ ions generally exhibit tighter errors than their formate adduct counterparts, while the adducts remain informative for confirming intact conjugation. Overall, the aqueous *T. erecta* extract is rich in acylated flavonol O-glycosides (notably derivatives of laricitrin, patuletin, and quercetagetin), multiple isorhamnetin-O-glucuronides, and early phenolic acids, with confirmed ellagic acid at low retention time. The combination of accurate mass agreement, predictable adduct/deprotonated behavior in formate, and reproducible fragment signatures provides a coherent chemical picture that is fully consistent with prior reports on *Tagetes* phenolics and with the known behavior of these classes in aqueous matrices [17,18,19,20].

The UPLC–QTOF–MS/MS analysis of the aqueous flower extract of *Tagetes erecta* revealed a profile strongly biased toward highly polar phenolics, as expected for a water-based matrix. Intact flavonol O-glycosides and O-acylglycosides dominate the chromatographic window, while organic and phenolic acids elute at the solvent front. Under formate-containing mobile phases, intact conjugates are frequently detected as formate adducts ([M+HCOO]^−^), whereas deprotonated molecules ([M−H]^−^) prevail for aglycones and low-molecular-weight acids; this duality yields characteristic adduct ↔ deprotonated pairs separated by ≈+46 u, which greatly facilitates class-level assignment. Early signals include quinic ([M−H]^−^ *m*/*z* 191 → 173/127/111) and gallic acid ([M−H]^−^ *m*/*z* 169 → 125), anchoring the hydrophilic end of the gradient. The core region (≈3.5–7.0 min) is populated by flavonol conjugates displaying diagnostic neutral losses (−162 u hexose; −86 u malonyl; −179/161 u caffeoyl) and resolving to aglycone anchors at *m*/*z* 317 (quercetagetin), 331 (patuletin), 301 (quercetin), and 285 (kaempferol). Multiple isorhamnetin-3-O-glucuronide isomers elute at RT ~6.07–6.50 min with [M−H]^−^ ≈ 491.24–491.28 and occasional [2M−H]^−^ at ≈983, in agreement with the literature on *Tagetes* flowers. High-mass clusters consistent with oligoglycosides are also evident; a robust adduct at *m*/*z* 864 supports a laricitrin tri-hexoside, while *m*/*z* 787 accompanied by the ladder 575 → 463 → 331 indicates a laricitrin di-hexoside carrying malonyl substitution. Notably, ellagic acid is confirmed at RT 2.07 min ([M−H]^−^ 300.9984; obs. ~301.000; −5.2 ppm), reflecting a modest but diagnostic ellagic/ellagitannin contribution. Across the dataset, [M−H]^−^ species show tighter mass errors than formate adducts; nevertheless, the adducts provide orthogonal confirmation of intact conjugation under aqueous, formate-rich conditions. Taken together, the aqueous extract is enriched in acylated flavonol O-glycosides (notably laricitrin/patuletin/quercetagetin derivatives), multiple isorhamnetin-O-glucuronides, and early phenolic acids. These observations are coherent with prior LC–MS characterizations of *Tagetes* phenolics and with the polarity-driven selectivity expected from water extraction [17,18,19,20].

### 2.2. AgNP Characterization

#### 2.2.1. Morphology

**Figure 1:**
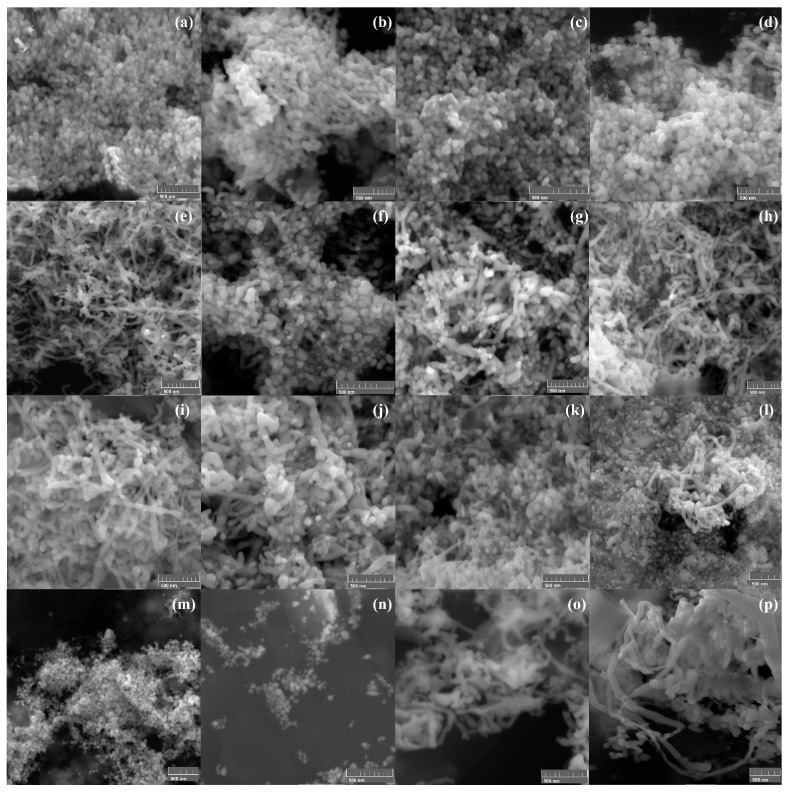
Selected SEM images of synthesized silver nanoparticles (AgNPs). Reference (R), gum Arabic (G), sodium chloride (S), talc powder (T): (**a**) R3, (**b**) R5, (**c**) R6, (**d**) R9, (**e**) R11, (**f**) R12, (**g**) G3, (**h**) G8, (**i**) G17, (**j**) S1, (**k**) S3, (**l**) S9, (**m**) T4, (**n**) T11, (**o**) T16, (**p**) T18.

**Figure 2:**
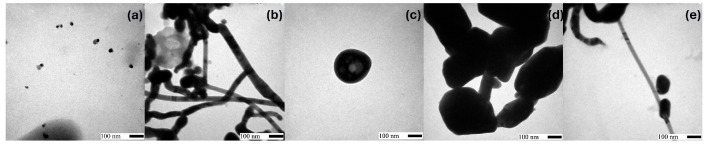
Selected TEM images. Reference (R), sodium chloride (S), talc powder (T), gum Arabic (G): (**a**) R6, (**b**) R11, (**c**) S6, (**d**) T11, (**e**) G11.

Selected AgNP SEM images are shown in Figure 1. Results indicate that without the use of additional agents and in the absence of stirring, spherical nanoparticles were synthesized (Figure 1a). Figure 1b–d show that although spherical nanoparticles are still the main product, some nanoribbons, rods, and wires were obtained when AgNO_3_ was previously stirred. Figure 1d shows a higher number of nanowires in comparison to Figure 1b,c due to a double stirring process. Stirring has previously been successfully used to modify the morphology of AgNPs [1]. The use of a higher volume of a more concentrated TEE (i.e., *Tagetes erecta* extract) (AgNO_3_/extract 1:3 ratio) implies a higher concentration of reducing agents, which favored the production of nanoribbons (Figure 1e). On the other hand, when the AgNO_3_/extract ratio was the opposite, sphere-like nanoparticles were obtained (Figure 1f). The effect of G (i.e., gum Arabic) can be seen in Figure 1g–i. In the absence of stirring (Figure 1g), a mixture of morphologies was produced. The number of nanoribbons and nanowires obtained in the presence of G increased (Figure 1h,i) when stirring was incorporated into the synthesis process. Regarding the use of S (i.e., sodium chloride) (Figure 1j–l), elongated morphologies can be observed even in the absence of stirring. Finally, irregular-shaped nanoparticles (Figure 1m,n) were obtained using T (i.e., talc powder) and either extract B (i.e., 10 g TE petals per 100 mL of deionized water) or stirring, turned into nanoribbons when T was used in combination with B extract and stirring (Figure 1o,p). Selected TEM images are shown in Figure 2. Nanospheres and nanoribbons synthesized without the use of any additional nucleating or structure-directing agent are shown in Figure 2a,b, respectively. Figure 2c shows an irregular-shaped nanoparticle with a AgCl nanoseed acting as a heterogeneous nucleus for metallic Ag. AgCl corresponds to the colorless section of the particle while the darker section corresponds to Ag [13]. Finally, Figure 2d,e show slightly elongated irregular-shaped nanoparticles and nanoribbons synthesized using T and G, respectively.

#### 2.2.2. Crystal Structure

**Figure 3:**
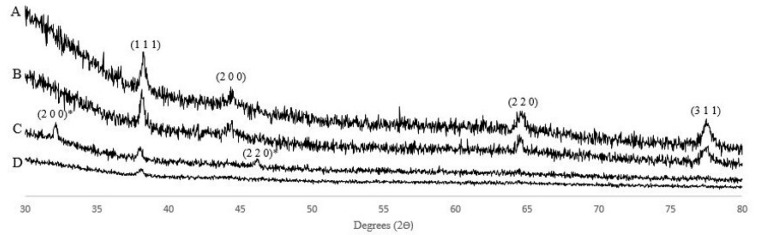
Selected XRD patterns of AgNPs. Reference (R), sodium chloride (S), talc powder (T), gum Arabic (G): (A) R11, (B) G17, (C) S6, (D) T4. * correspond to AgCl nucleus.

XRD patterns of the synthesized AgNPs are shown in Figure 3. Results show four major peaks located at 38.11°, 44.28°, 64.43°, and 77.47° corresponding to the diffraction of (111), (200), (220), and (311) planes of face-centered cubic metallic Ag [21]. The relatively high intensity of the (111) compared to the (200) and (220) peaks indicates that the samples had a preferential growth direction. Additionally, in the case of sample S6, peaks located at 32.23° and 46.24° corresponding to the (200) and (220) planes of the AgCl nucleus can be identified [22].

#### 2.2.3. Spectroscopic Analysis Results Are Shown in Figure 4

**Figure 4:**
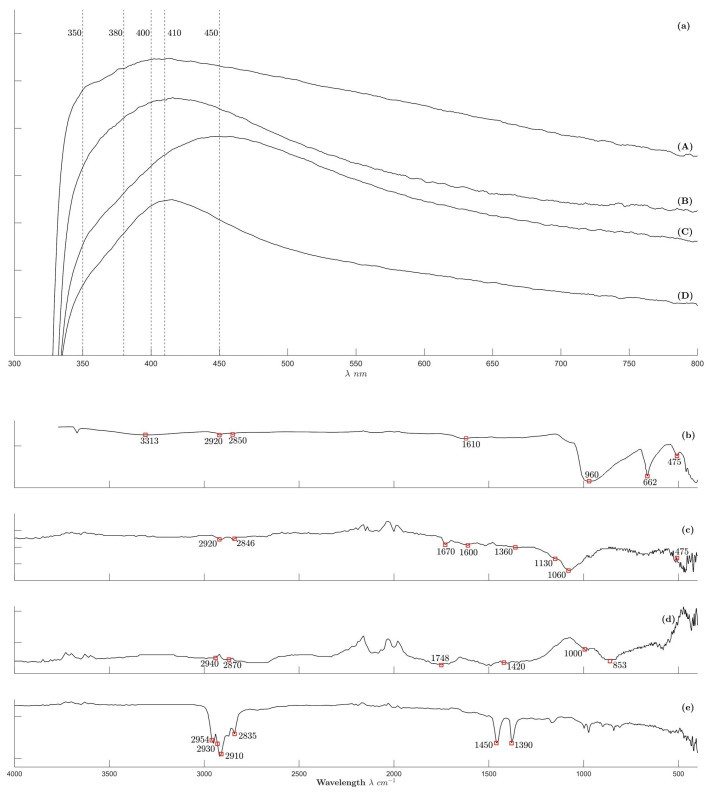
Spectroscopy analysis results: (**a**) UV–visible spectra: (A) S9, (B) G8, (C) R9, (D) T4. FTIR spectra: (**b**) (T11), (**c**) (R11), (**d**) (G11), (**e**) (S11).

UV–vis spectroscopy results are shown in Figure 4a. UV–vis was used to track the morphology of AgNPs, since differently shaped nanoparticles exhibit characteristic surface plasmon resonance bands at different frequencies. Samples S9 (Figure 4a-spectrumA) and G8 (Figure 4a-spectrumB) show a maximum absorption peak at 400 and 410 nm, respectively, indicating that nanoparticles are the main products. Additional subtle signals (i.e., shoulders) around 350 nm and 380 nm indicating the presence of elongated nanoparticles like nanoribbons and nanowires were observed in Figure 1lh. This type of shoulders has been previously observed and corresponds to the quadrupole resonance excitation and to the transverse plasmon resonance of elongated silver nanostructures, respectively [1,23]. Surface plasmon resonance absorption of anisotropic nanoparticles gives two or more absorption peaks due to the difference in their plasmon oscillations in the short and long axes. Figure 4a-spectrumC (R9) and spectrumD (T4) show that spherical nanoparticles are the main product, as indicated by a strong absorption peak around 410 and 450 nm, respectively. In all cases, a long tail over the wavelength around 400 nm to 800 nm is observed, indicating the presence of nanorods in all samples as well as the aggregation of the biosynthesized nanostructures [1,7,23,24,25,26].

FTIR spectra are shown in Figure 4b–e. Sample T11 (Figure 4b) shows peaks around 475 cm^−1^, 662 cm^−1^, 960 cm^−1^, and 1610 cm^−1^ corresponding to the Mg-O vibration, the Si-O bending, the Si-O-Si symmetrical stretching and the O-H bending, respectively, indicating the presence of T (i.e., talc powder), which also stabilizes AgNPs without chemical interaction. Peaks around 2939–2850 cm^−1^ correspond to (CH_2_, CH_3_ stretching) and 3313 cm^−1^ to alkenes stretching [27]. Sample (Figure 4c) R11 shows characteristic bands associated with AgNPs synthesized using TE extract. A peak near 475 cm^−1^ corresponds to the Mg-O vibration, while the stretching peaks of the C-N bond observed at 1060 cm^−1^ and 1130 cm^−1^ can be attributed to the presence of plant amines. A peak at 1360 cm^−1^ corresponds to phenolic groups (C-O stretching), while peaks around 1600–1670 cm^−1^ correspond to N-H and a peak at 2846 cm^−1^ and 2920 cm^−1^ to the C-H stretching [9,24]. Sample G11 is shown in Figure 4d. According to Venkatesham et al. [28], our results show characteristics peaks of G-AgNPs at 2940–2870 cm^−1^ (CH_2_, CH_3_ stretching), 1748 and 1000 cm^−1^ (asymmetric and symmetric stretching of COO^−^), 1420 cm^−1^ (bending of CH_2_, CH_3_), and 853 cm^−1^ (C-O stretch of ester). Finally, sample S11 (Figure 4e) shows peaks around 2954–2835 cm^−1^, 1450 cm^−1^, and 1390 cm^−1^ corresponding to the CH_2_ and CH_3_ stretching, bending and rocking; peaks around 2910 cm^−1^ and 2930 cm^−1^ (CH_2_ and CH_2_ stretching) are characteristic of Ag/AgCl natural extract-based nanoparticles [29].

**Figure 5:**
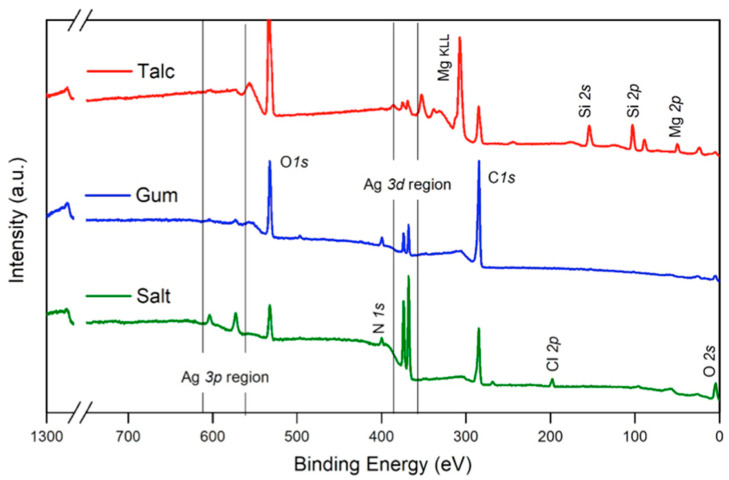
Wide-scan XPS spectra for samples containing T, G, and S, respectively. Main regions from 620 to 560 eV for Ag3*p* peak and from 380 to 360 eV for Ag3*d* are shown between lines.

Figure 5 shows wide-scan XPS spectra. Spectra peaks showing higher intensity correspond to C1*s*, O1*s*, and the Ag3*d* regions. Additional peaks corresponding to N1*s*, Ag3*p*, Cl2*p*, and Si2*p* are due to the use of additional compounds during the process reaction (i.e., nucleating or structure-director agents). The regions showing the presence of Ag for core levels 3*d* and 3*p* are presented as doublets and correspond to its two main cross sections. Regarding the use of S, it can be observed that the signals corresponding to Ag3*d* and Ag3*p* are clearly more intense than those registered when G or T were used, indicating the presence of a higher Ag content.

**Figure 6:**
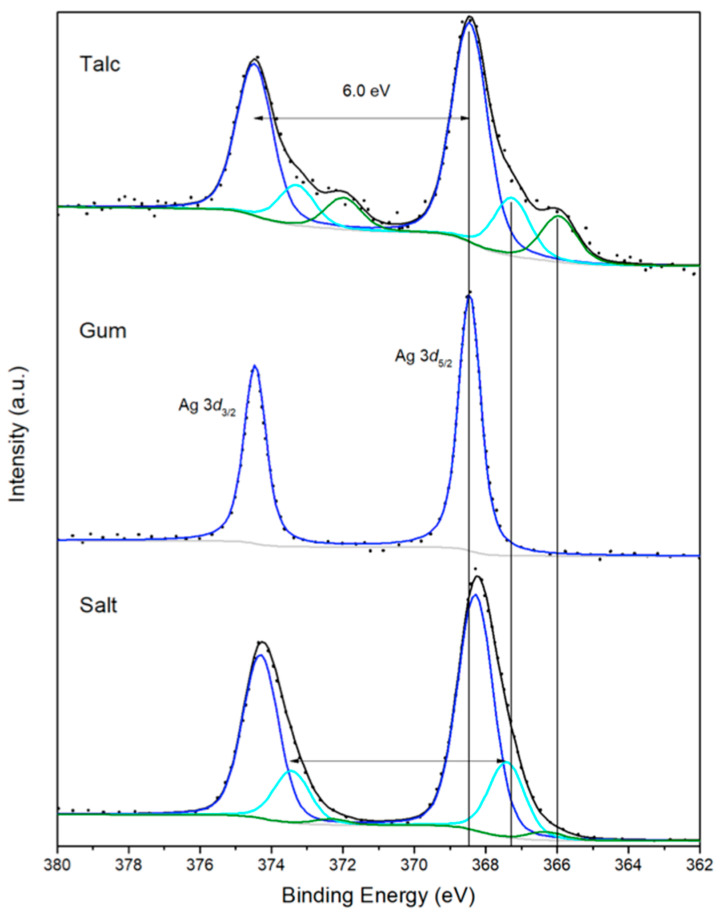
XPS high-resolution Ag3*d* spectra for samples using T, G, and S, respectively.

Additionally, Figure 6 shows XPS high-resolution spectra of the Ag3*d* region for samples using T (talc powder), G (gum Arabic), and S (salt). The signal is deconvoluted with doublets that correspond to 6.0 eV of spin-orbital splitting and the peak area ratio of orbital *d* and coupling *3/2* and *5/2*. The main peak at the position of 368.5 eV is attributed to the metallic particles in natural form, while the peak at 367.4 eV corresponds to Ag_2_O and the peak around 366.0 eV is assigned to a higher oxidation state which may naturally occur for AgNPs synthesized in ambient conditions and/or silver species with a higher oxidation state that could be located at the ends of the nanoparticles formed during biosynthesis.

Finally, Table 3 shows the atomic content for samples using T (talc powder), G (gum Arabic), and S (salt). It can be noticed that the sample using S shows the highest Ag content. Other chemical elements such as silicon, magnesium, nitrogen, and chloride are present due to the use of nucleating or structure-director agents in the biosynthesis of AgNPs. A high Ag content may have a positive effect on the antibacterial activity of the biosynthesized AgNPs.

### 2.3. Antibacterial Activity

**Figure 7:**
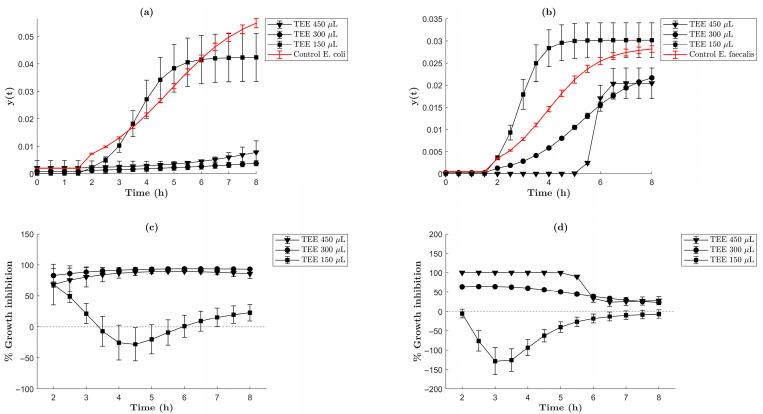
Curves of the growing parameter (y_(t)_) and percentage of growth inhibition of *E. faecalis* and *E. coli* bacteria. (**a**) Comparative graph of (y_(t)_) *E. coli* bacteria. (**b**) Comparative graph of (y_(t)_) of *E. faecalis* bacteria. These graphs show the growth behavior of the bacteria under ideal conditions (positive control) and how the growth parameter was modified in the presence of TEE (i.e., *Tagetes erecta* extract). (**c**) Comparative graph of % growth inhibition of *E. coli* bacteria due to TEE. (**d**) Comparative graph of % growth inhibition of *E. faecalis* bacteria due to TEE.

**Figure 8:**
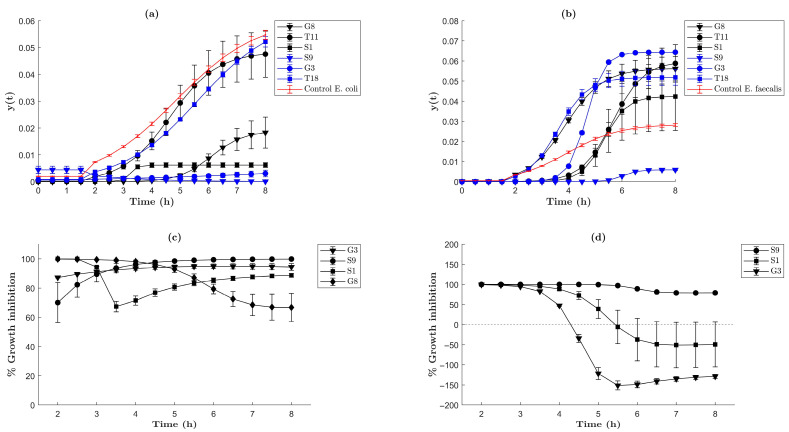
Curves of the growing parameter (y_(t)_) and percentage of growth inhibition of *E. faecalis* and *E. coli* bacteria. (**a**) Comparative graph of (y_(t)_) *E. faecalis* bacteria. (**b**) Comparative graph of (y_(t)_) of *E. coli* bacteria. These graphs show the growth behavior of the bacteria under ideal conditions (positive control) and how its growth parameter was modified in the presence of the nanomaterial suspensions. (**c**) Comparative graph of % growth inhibition of *E. coli* bacteria. (**d**) Comparative graph of % growth inhibition of *E. faecalis* bacteria. These graphs show the % growth inhibition for the most effective nanomaterials.

In this work, bacterial growth was monitored by means of the optical density method (OD595). Samples were taken every 30 min. Results are shown in Figure 7 and Figure 8 for TEE (i.e., *Tagetes erecta* extract) and AgNPs, respectively. It was observed that TEE showed an inhibitory effect of about 90% during the first 7 h against *E. coli* (Figure 7a,c) when 300 µL or 450 µL of extract was used. After 7 h, a gradual increase in bacterial growth was observed. On the other hand, for *E. faecalis* (Figure 7b), the addition of 300 µL decreased bacterial growth up to 62.65% during the first 3.5 h, while the addition of 450 µL registered a 99.99% inhibitory effect during the first 5 h (Figure 7d). After that time, in both experiments (using 300 µL or 450 µL of TEE), the inhibitory effect decreased to 23.08%, which caused the growth of *E. faecalis* to increase up to similar values to those of the positive control.

The antibacterial activity of six different AgNP suspensions (T18, G3, S9, S1, T11, and G8) was evaluated. Samples S1, S9, and G3 showed inhibitory activity against *E. coli* and *E. faecalis* bacteria. As observed in Figure 8a, samples S1, S9, G3, and G8 showed *E. coli* growth inhibition. However, only S9 and G3 showed higher efficiency (90–99%) as observed in Figure 8c. On the other hand, samples S9, S1, and T11 showed antimicrobial properties against *E. faecalis*. As indicated in Figure 8b, during the first 3 h, 90–100% inhibition was observed. But, after 3 h, only S9 showed a constant inhibition for up to 8 h (Figure 8d). AgNPs synthesized using S and G as additional agents showed a higher Ag content, which could be the reason for more effective antibacterial activity as indicated by these results.

The biosynthesis of AgNPs using plant extracts is a procedure that includes the interaction of solutions of silver nitrate (AgNO_3_) with the biomolecules present in plant extracts such as amino acids, proteins, alkaloids, flavonoids, polyphenols, enzymes, tannins, carbohydrates, and saponins [30]. Taking this information into consideration, it is essential to carry out a qualitative analysis to identify the main groups of biomolecules present in the TEE that could have functioned as reducing agents in the reduction of Ag^+^ ions to AgNPs. However, despite having identified the presence of molecules that have OH groups susceptible to oxidation, the TEE/AgNO_3_ reaction was unusually fast compared to what was observed in previous synthesis processes. Right after the TEE addition, a black-gray color appeared, indicating the reduction of silver ions dispersed in the medium. This behavior could be due to the presence of other metallic ions dispersed in the TEE.

TEE (i.e., *Tagetes erecta* extract) showed different bacteriostatic activity against *E. coli* and *E. faecalis.* Volumes between 300 and 450 µL of TEE showed the maximum percentage of inhibition (89–93%) against *E. coli*. However, the inhibitory properties against the *E. faecalis* bacteria were less effective. Most Gram-positive bacteria have a relatively thick continuous cell wall, which is composed largely of peptidoglycan. In contrast, the peptidoglycan layer in Gram-negative bacteria is thin. This suggests that the metabolites present in the TEE could have more affinity to the thin continuous cell wall of *E. faecalis* and consequently did not move easily across the membrane or it was easier for the metabolites to cross the inner and outer membranes of *E. coli*. In both cases, it is necessary to have at least 300 µL of TEE per 10 mL of LB media to evidence inhibitory properties. As for the mechanism by which AgNPs break down the membrane permeability barrier, AgNPs may perturb the membrane lipid bilayers, causing the leakage of ions and other materials as well as forming pores and dissipating the electrical potential of the membrane [31]. Zheng et al. suggest that the more the AgNPs’ size is reduced, the higher surface to volume ratio these AgNPs would possess, which, in turn, would also allow the AgNPs to better interact with the bacteria [32].

In addition, the presence of sodium chloride during the process of synthesis of AgNPs could interact with Ag^+^ ions to form silver chloride [33,34]. Gupta, A. et al. suggested that the kinetics of dissolution are strongly dependent on the Cl/Ag ratio and can be interpreted using the thermodynamically expected speciation of Ag in the presence of chloride [35]. They also showed that the toxicity of AgNPs to *E.coli* at various Cl(−) concentrations is governed by the amount of dissolved AgCl_x_^(x−1)−^ species, suggesting an ion effect rather than a nanoparticles effect [35]. Although it has been demonstrated that the presence of AgNPs can have an inhibitory effect, when comparing materials S9, G3, and T18, which have the same promoting amount of Ag(0) and which, theoretically would be expected to have similar inhibitory activity, it was observed that sample S9 had a greater inhibitory potential for both bacterial strains than that observed for materials G3 and T18. Likewise, when comparing the inhibitory activity between materials S1 and S9, where the difference lies in the initial volume of AgNO_3_, it was observed that material S1 is not as effective in terms of inhibitory action as S9 [36].

## 3. Materials and Methods

### 3.1. Materials

AgNO_3_ (Sigma-Aldrich, St. Louis, MO, USA), S (Karal), T (Sigma-Aldrich), G (Sigma-Aldrich). All chemicals were used as received. TE plants were bought from a nursery in Guadalajara, Jalisco, Mexico. Flowers were washed with water and later petals were separated and washed with deionized water before extract preparation.

### 3.2. TEE Preparation

Two different TEEs were prepared by boiling 5 g (extract A) or 10 g (extract B) of previously washed TE petals per 100 mL of deionized water for 30 min. Solutions were filtered to remove unwanted residues.

### 3.3. Synthesis of AgNPs

TEE as a source of reducing agents, AgNO_3_ as a metal precursor, and a nucleating agent (i.e., S) or a structure-director agent (i.e., G or T) were used. Four different groups of AgNPs, each one consisting of 18 samples, were synthesized: group 1 (reference samples R1 to R18), group 2 using G (G1 to 18), group 3 using S (S1 to S18), and group 4 using T (T1 to T18). Reactions were performed in an autoclave at 120 °C for 15 min according to Table 4. Samples were washed using deionized distilled water and acetone and centrifuged at 4000 rpm for 7 min to eliminate reaction residues.

### 3.4. Phytochemical Screening of TEE

Phytochemical screening of TEE was performed for various metabolites by means of different assays: saponin (hot water and Rosenthaler), triterpene (chloroform/H_2_SO_4_), tannins (chloride ferric salt), flavonoid (ammonia vapors, Shinoda, and NaOH), and coumarin (NaOH/heat/UV light) tests [37,38]. All experiments were performed in triplicate.

#### 3.4.1. Saponins

##### Hot Water Test

To perform this test, 1 mL of TEE and 10 mL of distilled water were added to a test tube, which was heated for 30 min in a water bath. In the second stage, the tube was shaken vigorously for 3 min. The formation of stable honeycomb-like foam is considered a positive result.

##### Rosenthaler Assay

In total, 1 mL of TEE, a drop of Rosenthaler reactive, and a drop of sulfuric acid (97%) were added into the assay tube. The formation of violet color is considered a positive test for pentacyclic triterpene saponins.

#### 3.4.2. Triterpenes

In total, 1 mL of TEE and 1 mL of chloroform were added into an assay tube. Subsequently, 1 mL of acetic anhydride was added and the tube was left to cool to room temperature. Finally, two drops of sulfuric acid (97%) were added. If colors red, pink, green, purple, or blue are identified, it is considered a positive test.

#### 3.4.3. Tannins

In total, 1 mL of TEE was diluted to a final volume of 25 mL with distilled water. Then, the solution was filtered and an aliquot (1 mL) of this dilution was added into an assay tube with 1 mL of a saturated solution of ferric chloride salt. The presence of red precipitate is considered a positive test.

#### 3.4.4. Flavonoids

##### Ammonia Vapors

In total, 1 mL of TEE was dried and diluted with ethanol (97%). A piece of paper was impregnated with this solution and left to dry. Finally, the impregnated paper was indirectly exposed to ammonia vapor (30%). The presence of ochre yellow color is considered a positive test.

##### Shinoda

In total, 1 mL of TEE was diluted with 1 mL of distilled water. After that, 0.001 g of magnesium powder and 2 drops of chloride acid (37%) were added. The appearance of colors ranging from deep red to magenta indicates the presence of flavanone or dihydroflavonol. Dihydrochalcones and other flavonoids do not react.

##### NaOH Assay

In total, 1 mL of TEE was diluted with 1 mL of distilled water in an assay tube. Subsequently, 3 drops of a diluted NaOH solution were added. The appearance of yellow or orange colors indicates the presence of flavonoids.

#### 3.4.5. Coumarins

The presence of coumarins was detected by heating 1 mL of TEE placed into an assay tube and covered with filter paper impregnated with an alkaline solution. The presence of fluorescent points observed under a UV lamp is considered a positive test.

#### 3.4.6. Quantification of Reducing Sugars in TEE

The presence of available reducing sugars in TEE was quantified before and after the synthesis of AgNPs by a modified method of the Fehling reaction [39]. Control sample: In total, 5 mL of TEE was diluted to a final volume of 25 mL with distilled water (Solution C.a). An aliquot of C.a (10 mL) was taken and diluted to a final volume of 100 mL (Solution C.b). In triplicate, 10 mL of C.b, 5 mL of Fehling A, 5 mL of Fehling B, 50 mL of distilled water, and five drops of Methylene Blue as indicator were added in a 250 mL Erlenmeyer flask. The mixture was heated (60–70 °C) and titrated with glucose solution (0.5% glucose, *w*/*v*) until the characteristic turning point of the technique (i.e., red) was observed. Experimental samples: Samples were tested by taking 5 mL of the supernatant right after the synthesis and were diluted to a final volume of 25 mL with distilled water (Solution I.a). A total of 10 mL of I.a was taken and diluted to a final volume of 100 mL (Solution I.b). In triplicate, 10 mL of I.b, 5 mL of Fehling A, 5 mL of Fehling B, 50 mL of distilled water, and five drops of Methylene Blue as indicator were added into a 250 mL Erlenmeyer flask. The mixture was heated (60–70 °C) and titrated with glucose solution until the turning point characteristic of the technique appeared. The difference between the volumes of glucose titration of the samples before and after the synthesis indicates the chemical equivalents of the reducing agent used to reduce silver.

#### 3.4.7. Quantification of Fe^2+^ in TEE

The presence of available reducing Fe^2+^ in TEE was quantified (before and after the synthesis of silver nanoparticles) by a modified method [40]. Control sample: In triplicate, 5 mL of TEE was diluted to a final volume of 100 mL with distilled water. In total, 10 mL of the dilution, 2 mL of H_2_SO_4_ (97%), and two drops of diphenylamine as indicator were added into a 250 mL Erlenmeyer flask. The mixture was titrated with potassium dichromate solution (K_2_Cr_2_O_7_, 12 mM) until the turning point characteristic of the technique (blue-violet) was observed.

Experimental samples: In total, 5 mL was taken from the supernatant after synthesis and diluted to a final volume of 100 mL with distilled water. In triplicate, 10 mL of this dilution, 2 mL of H_2_SO_4_ (97%), and two drops of diphenylamine as indicator were added in a 250 mL Erlenmeyer flask. The mixture was titrated with K_2_Cr_2_O_7_ solution until the turning point characteristic of the technique (blue-violet) was observed. The difference between the volumes of K_2_Cr_2_O_7_ (before and after the synthesis) indicates the chemical equivalents of the Fe^2+^ used to reduce silver.

### 3.5. TEE UPLC-Qtof-MS/MS Profile

UPLC-Qtof-MS/MS analysis was performed using a Waters Acquity UPLC H-Class system (Milford, MA, USA). The separation was carried out in a Waters Acquity UPLC BEH C18 (2.1 × 100 mm, 1.7 μm) column at 40 °C, using a gradient of solvent A (water-formic acid 0.05% *v*/*v*) and solvent B (acetonitrile-formic acid 0.05% *v*/*v*); the gradient was 0–1 min (95% A), 1–11 min (95–30% A), 11–12 min (30–95% A), and 12–15 min (95% A), the flow was 0.3 mL/min, and the injection volume was 10 μL. Mass spectrometry was performed in negative mode in a Waters Xevo G2-XS QTof spectrometer (Waters Corporation Milford, Massachusetts, USA) with electrospray ionization (ESI); the parameters were cone voltage of 40 kV, a capillarity voltage of 2.5 kV, a font temperature of 100 °C, desolvation temperature of 250 °C, and collision energy of 6 eV. The mass range was from 50 to 1000 *m*/*z*.

### 3.6. AgNP Characterization

AgNPs were characterized by means of scanning electron microscopy (SEM) and transmission electron microscopy (TEM) using a TESCAN MIRA-3 LMU (TESCAN, Brno, Czech Republic) operating at 20 kV and a JEOL JEM1010 microscope (JEOL Ltd., Tokyo, Japan) operating at 80 kV, respectively, to analyze the morphology of the biosynthesized AgNPs and to evaluate the effect of the use of nucleating or structure-director agents. A Panalytical-Empyrean device was used for X-ray diffraction (XRD) with Cu radiation operating at 40 kV and 30 mA to identify the crystalline structure of the AgNPs. Ultraviolet–visible spectroscopy (UV–vis) spectra were recorded using a Genesys 10S device (Thermo Fisher Scientific, Waltham, MA, USA) scanning from 300 to 1100 nm to track the morphology of AgNPs. Fourier transform infrared spectroscopy (FTIR) tests were performed using a Nicolet iS50 spectrophotometer (Thermo Fisher Scientific) over a range of 400 to 4000 nm to identify the characteristic bands associated with biosynthesized AgNPs. Chemical analysis of samples biosynthesized using additional agents (G, S, or T) was performed by means of X-ray photoelectron spectroscopy (XPS) tests in order to analyze the final composition of the samples due to the presence of such additional agents. Samples prepared as films were measured using an X-ray photoelectron analyzer (Phoibos 150, SPECS, Berlin, Germany) with a monochromatic Al K_α_ (hν = 1486.7 eV) XR50M source operated at 250 W. High-resolution spectra were obtained using a step size of 0.1 eV and pass energy of 10 eV. During the analysis, the main chamber pressure was maintained at 1.3 × 10^−9^ mbar and a flood gun current of 2.5 eV for charge compensation due to the insulated substrate. Wide-scan spectra data of the main core levels are presented. The corresponding peak areas were determined using the software AAnalyzer v2.25 to calculate the atomic concentration [41,42]. The corresponding chemical shift using reference position at 285.0 eV for main *C–C* bonding was obtained using simultaneous fitting for C*1s* [43].

### 3.7. Antibacterial Activity

Growth inhibition assays were performed against Gram-positive (*E. faecalis*) and Gram-negative bacteria (*E. coli*). Overnight cultures of *E. faecalis* and *E. coli* were prepared in LB broth media under agitation (250 rpm) at 37 °C for 12 h. Bacterial growth was monitored by measuring the optical density at 595 nm (OD_595_).

To evaluate the inhibition growth of the selected bacterial cultures, samples of 2 μL taken from the overnight cultures were mixed with 10 mL of LB media culture and deposited into sterile tubes. In triplicate, samples of TEE (150 µL, 300 µL, and 450 µL) were tested, as well as different nanomaterial suspensions (100 µL of each sample). The distribution of the tests is shown in Table 5.

All experiments were performed in triplicate and the average of three independent experiments was calculated for each assay using Equation (1). Length of the lag phase (λ), growth rate represented by the maximum slope (μ), and the maximum cell growth (A) appear in Equation (1), in which t indicates the growing period and y_(t)_ indicates the growing parameter [44].

(1) $$y_{\left(t\right)}=\frac{A}{1+exp\left(\left(\frac{4\mu }{A}\right)\left(\lambda -1\right)+2\right)}$$

Growth inhibition % was calculated considering the growth in the absence of any test material as 100% according to Equation (2) [45].

(2) $$Growth\;inhibition\;\%=100-\left[\frac{OD_{\left(test\;sample\right)}-OD_{\left(Blank\right)}}{OD_{\left(control\right)}-OD_{\left(Blank\right)}}\right]\times 100$$

## 4. Conclusions

A simple and environmentally friendly approach to modify the morphology of AgNPs using non-toxic agents is presented. The results indicate that S, G, and T were successfully used to modulate AgNP morphology. The use of G favored the formation of nanowires and nanoribbons, while S promoted the generation of more elongated particles and T led to the formation of nanoribbons as a product of the synthesis. SEM and TEM analyses showed that differently sized and shaped nanoparticles (e.g., particles, rods, wires) were obtained when a heterogeneous nucleating agent (S) and structure-director agents (G and T) were used. UV–vis spectroscopy confirmed the presence of AgNPs with different morphologies through the appearance of characteristic absorption peaks. XRD patterns were consistent with previously reported data for morphologies such as spheres, wires, and rods. XPS analysis indicated that particles showing different Ag content were obtained due to the use of S, G, and T, which directly influenced their antibacterial performance. AgNPs obtained using S exhibited the highest antibacterial activity against *E.coli* and *E. faecalis*.

## Figures and Tables

**Table 1:** Results of phytochemical screening test of TEE.

Phytochemical Compounds	Test	Result
Saponins	Hot water	Honeycomb stable for about 60 min. Positive test
	Rosenthaler assay	Negative
Triterpenes	Chloroform/H_2_SO_4_	Negative
Tannins	Chloride ferric salt	Negative
Flavonoids	Ammonia vapors	Positive for the presence of flavonoids
	Shinoda	Positive for the presence of flavanone or dihydroflavonol
	NaOH Assay	Positive for the presence of flavonoids
Coumarins	NaOH/heat/UV light	Negative

**Table 2:** Compounds identified in the *T. erecta* total aqueous extract using UPLC–QTOF–MS/MS.

No.	RT (min)	*m*/*z* Observed	Ion Compared	*m*/*z* Theoretical	Error (ppm)	Neutral Formula	Compound	Major Fragments (%)	Reference
1	0.820	191.117	[M−H]^−^	191.055563	−0.061437 u; −321.6 ppm	C_7_H_12_O_6_	Quinic acid	191 (base); 173, 127, 111	Parejo et al., 2004 [20]
2	1.097	191.080	[M−H]^−^	191.055563	−0.024437 u; −127.9 ppm	C_7_H_12_O_6_	Quinic acid (co-eluting isomer)	191 (base); 173, 127, 111	Parejo et al., 2004 [20]
3	1.320	169.079	[M−H]^−^	169.013698	−0.065302 u; −386.4 ppm	C_7_H_6_O_5_	Gallic acid	169 → 125 (CO_2_ loss)	Parejo et al., 2004 [20]
4	2.070	301.000	[M−H]^−^	300.998442	−0.001558 u; −5.2 ppm	C_14_H_6_O_8_	Ellagic acid	301 (base); 257, 229	Fracassetti et al., 2013 [17]
5	3.140	645.135	[M+HCOO]^−^	645.1456	+0.010600 u; +16.4 ppm	C_29_H_28_O_14_	Kaempferol-3-O-(6″-galloyl)-hexoside	645 (adduct), ~599 ([M−H]^−^), 453 (−Hex), 437 (−acyl), 285 (aglycone), 169/125 (galloyl)	Parejo et al., 2004 [20]
6	3.790	687.139	[M+HCOO]^−^	687.119739	−0.019261 u; −28.0 ppm	C_30_H_26_O_16_	Quercetagetin-O-caffeoyl-hexoside	687 (100); 641; 317; 179/161	Parejo et al., 2004 [20]
7	4.360	555.158	[M+HCOO]^−^	555.1449	+0.013100 u; +23.6 ppm	C_23_H_22_O_14_	Flavonol-O-glycoside	509 ([M−H]^−^), 345 (aglycone, −Hex), 327/315 (H_2_O/CO), 179/161 (caffeoyl, trace)	Parejo et al., 2004 [20]
8	4.680	525.160	[M+HCOO]^−^	525.088045	−0.071955 u; −137.0 ppm	C_21_H_20_O_13_	Myricetin-3-O-hexoside	525 (adduct); 479 ([M−H]^−^); aglycone 317	Moliner et al., 2018 [19]; Burlec et al., 2021 [18]
9	5.090	539.168	[M+HCOO]^−^	539.103695	−0.064305 u; −119.3 ppm	C_22_H_22_O_13_	Patuletin-O-hexoside	539 (adduct); 493 ([M−H]^−^); aglycone 331	Parejo et al., 2004 [20]; Burlec et al., 2021 [18]
10	5.450	579.201	[M+HCOO]^−^	579.098610	−0.102390 u; −176.8 ppm	C_24_H_22_O_14_	Kaempferol-3-O-(6″-malonyl)-hexoside (putative)	579 (adduct); 245/305	Parejo et al., 2004 [20]
11	5.570	317.084	[M−H]^−^	317.029742	−0.054258 u; −171.1 ppm	C_15_H_10_O_8_	Quercetagetin (aglycone)	317; [2M−H]^−^ ≈ 635	Parejo et al., 2004 [20]
12	5.680	864.331	[M+HCOO]^−^	863.209342	−1.121658 u; −1299.4 ppm	C_34_H_42_O_23_	Laricitrin-trihexoside (putative)	864 adduct series; 317/331 present	Moliner et al., 2018 [19]; Burlec et al., 2021 [18]
13	6.070	491.244	[M−H]^−^	491.082566	−0.161434 u; −328.7 ppm	C_22_H_20_O_13_	Isorhamnetin-3-O-glucuronide (co-eluting)	491; [2M−H]^−^ ≈ 983 (weak)	Burlec et al., 2021 [18]
14	6.070	545.151	[M−H]^−^	545.150645	−0.000355 u; −0.7 ppm	C_23_H_30_O_15_	Flavonol-O-acyl-glucuronide (putative)	Formate adduct ~591	Parejo et al., 2004 [20]
15	6.260	491.245	[M−H]^−^	491.082566	−0.162434 u; −330.8 ppm	C_22_H_20_O_13_	Isorhamnetin-3-O-glucuronide (isomer)	491; [2M−H]^−^ ≈ 983	Burlec et al., 2021 [18]
16	6.400	625.333	[M+HCOO]^−^	625.104089	−0.228911 u; −366.2 ppm	C_25_H_24_O_16_	Laricitrin-3-O-(6″-malonyl)-hexoside (putative)	625 (adduct) ↔ 579 ([M−H]^−^); 491 co-eluting	Parejo et al., 2004 [20]; Burlec et al., 2021 [18]
17	6.400	491.246	[M−H]^−^	491.082566	−0.163434 u; −332.8 ppm	C_22_H_20_O_13_	Isorhamnetin-3-O-glucuronide (co-eluting)	491; [2M−H]^−^ ≈ 983 (trace)	Burlec et al., 2021 [18]
18	6.500	491.280	[M−H]^−^	491.082566	−0.197434 u; −402.0 ppm	C_22_H_20_O_13_	Isorhamnetin-3-O-glucuronide (isomer)	491; 534/535; 580/625	Burlec et al., 2021 [18]
19	6.650	787.243	[M+HCOO]^−^	787.156912	−0.086088 u; −109.4 ppm	C_31_H_34_O_21_	Laricitrin-dihexoside-malonyl	787 adduct; 575→463→331	Parejo et al., 2004 [20]; Moliner et al., 2018 [19]
20	6.730	485.405	[M−H]^−^	493.1135	+7.7085 u; +15,630 ppm	C_22_H_22_O_13_	Laricitrin-3-O-hexo-side	331/330/329 (aglycone ladder), 314 (demethylation), 483/489	Moliner et al., 2018 [19]; Burlec et al., 2021 [18]

Error reported as (theoretical—observed).

**Table 3:** Atomic content detected by XPS.

Core Level	Talc	Gum % Atomic Presence	Salt
Carbon 1*s*	19.6	72.8	60.2
Oxygen 1*s*	35.8	22.6	17.3
Silver 3*d*	1.0	1.7	12.9
Silicon 2*p*	23.4	-	-
Magnesium 2*p*	20.2	-	-
Others (N1*s*, Cl2*p*)	-	2.9	9.6

**Table 4:** Preparation conditions of AgNPs.

Sample Number	AgNO_3_ [20 mM]	Stirring	* Additional Reaction Agent [0.01 M]	Extract (mL)	TEE	Stirring
1	3 mL	x	3 mL	3 mL	A	
2	1.5 mL		3 mL	4.5 mL	A	
3	4.5 mL		3 mL	1.5 mL	A	
4	3 mL	30 min	3 mL	3 mL	A	
5	1.5 mL	30 min	3 mL	4.5 mL	A	
6	4.5 mL	30 min	3 mL	1.5 mL	A	
7	3 mL	30 min	3 mL	3 mL	A	30 min
8	1.5 mL	30 min	3 mL	4.5 mL	A	30 min
9	4.5 mL	30 min	3 mL	1.5 mL	A	30 min
10	3 mL		3 mL	3 mL	B	
11	1.5 mL		3 mL	4.5 mL	B	
12	4.5 mL		3 mL	1.5 mL	B	
13	3 mL	30 min	3 mL	3 mL	B	
14	1.5 mL	30 min	3 mL	4.5 mL	B	
15	4.5 mL	30 min	3 mL	1.5 mL	B	
16	3 mL	30 min	3 mL	3 mL	B	30 min
17	1.5 mL	30 min	3 mL	4.5 mL	B	30 min
18	4.5 mL	30 min	3 mL	1.5 mL	B	30 min

* Depending on the group of samples biosynthesized, no additional agent (group 1—R) or only one additional agent [group 2 (gum Arabic, G), group 3 (salt, S), or group 4 (talc, T)] was used in the synthesis. In all cases, AgNO_3_ was used as a metal precursor and TEE (A-5 g/100 mL or B-10 g/100 mL) as a source of reducing and stabilizing agents.

**Table 5:** Distribution of tests used to evaluate the antimicrobial activity of TEE and different nanomaterials.

Test	*E. faecalis*	*E. coli*
Blank	10 mL LB media	10 mL LB media
Positive control	10 mL LB media + 2 μL of the overnight	10 mL LB media + 2 μL of the overnight
Blank I	10 mL LB media + 100 μL AE-Tef	10 mL LB media + 100 μL AE-Tef
TEE I	10 mL LB media + 2 μL of the overnight + 100 μL AE-Tef	10 mL LB media + 2 μL of the overnight + 100 μL AE-Tef
Blank II	10 mL LB media + 450 μL AE-Tef	10 mL LB media + 450 μL AE-Tef
TEE II	10 mL LB media + 2 μL of the overnight + 450 μL AE-Tef	10 mL LB media + 2 μL of the overnight + 450 μL AE-Tef
Blank III	10 mL LB media + 100 μL nanomaterial	10 mL LB media + 100 μL nanomaterial
Nanomaterial test	10 mL LB media + 2 μL of the overnight + 100 μL nanomaterial	10 mL LB media + 2 μL of the overnight + 100 μL nanomaterial

## Data Availability

Data is available on request. Contact E-mail: edgar.lopezn@academicos.udg.mx.

## References

[1] Gómez-Acosta A., Manzano-Ramírez A., López-Naranjo E.J., Apatiga L.M., Herrera-Basurto R., Rivera-Muñoz E.M. (2015). Silver nanostructure dependence on the stirring-time in a high-yield polyol synthesis using a short-chain PVP. Mat. Lett..

[2] Padalia H., Moteriya P., Chanda S. (2015). Green synthesis of silver nanoparticles from marigold flower and its synergistic antimicrobial potential. Arab. J. Chem..

[3] Ahmed S., Ahmad M., Swami B.L., Ikram S. (2016). A review on plants extract mediated synthesis of silver nanoparticles for antimicrobial applications: A green expertise. J. Adv. Res..

[4] Lomelí-Rosales D.A., Zamudio-Ojeda A., Reyes-Maldonado O.K., López-Reyes M.E., Basulto-Padilla G.C., López-Naranjo E.J., Zuñiga-Mayo V.M., Velázquez-Juárez G. (2022). Green synthesis of gold and silver nanoparticles using leaf extract of Capsicum chinense plant. Molecules.

[5] Rafique M., Sadaf I., Rafique M.S., Tahir M.B. (2016). A review on green synthesis of silver nanoparticles and their applications. Artif. Cells Nanomed. Biotechnol..

[6] Wu L., Wu W., Jing X., Huang J., Sun D.D., Odoom-Wubah T., Liu H., Wang H., Li Q. (2013). Trisodium citrate-assisted biosynthesis of silver nanoflowers by Canarium album foliar broths as a platform for SERS detection. Ind. Eng. Chem. Res..

[7] Logaranjan K., Jaculin Raiza A., Gopinath S.C.B., Chen Y., Pandian K. (2016). Shape- and size-controlled synthesis of silver nanoparticles using Aloe vera plant extract and their antimicrobial activity. Nanoscale Res. Lett..

[8] Smirnov O., Dzhagan V., Kovalenko M., Gudymenko O., Dzhagan V., Mazur N., Isaieva O., Maksimenko Z., Kondratenko S., Skoryk M. (2023). ZnO and AgNP-decorated ZnO nanoflowers: Green synthesis using Ganoderma lucidum aqueous extract and characterization. RSC Adv..

[9] Katta V.K.M., Dubey R.S. (2021). Green synthesis of silver nanoparticles using Tagetes erecta plant and investigation of their structural, optical, chemical and morphological properties. Mater. Today Proc..

[10] Maji A., Beg M., Das S., Aktara M.N., Patra A., Islam M.M., Hossain M. (2020). Study on the antibacterial activity and interaction with human serum albumin of Tagetes erecta inspired biogenic silver nanoparticles. Process Biochem..

[11] Qi X., Balankura T., Zhou Y., Fichthorn K.A. (2015). How structure-directing agents control nanocrystal shape: PVP-mediated growth of Ag nanocubes. Nano Lett..

[12] Samanta S., Pyne S., Sarkar P., Sahoo G.P., Bar H., Bhui D.K., Misra A. (2010). Synthesis of silver nanostructures of varying morphologies through seed mediated growth approach. J. Mol. Liq..

[13] Schuette W.M., Buhro W.E. (2013). Silver chloride as a heterogeneous nucleant for the growth of silver nanowires. ACs Nano.

[14] Bruna T., Maldonado-Bravo F., Jara P., Caro N. (2021). Silver nanoparticles and their antibacterial applications. Int. J. Mol. Sci..

[15] Ahmad S.A., Das S.S., Khatoon A., Ansari M.T., Afzal M., Hasnain M.S., Nayak A.K. (2020). Bactericidal activity of silver nanoparticles: A mechanistic review. Mater. Sci. Energy Technol..

[16] Menichetti A., Mavridi-Printezi A., Mordini D., Montalti M. (2023). Effect of size, shape and surface functionalization on the antibacterial activity of silver nanoparticles. J. Funct. Biomater..

[17] Fracassetti D., Cost C., Moulay L., Tomás-Barberán F.A. (2013). Ellagic acid derivatives, ellagitannins, proanthocyanidins and other phenolics, vitamin C and antioxidant capacity of two powder products from camu-camu fruit (*Myrciaria dubia*). Food Chem..

[18] Burlec A.F., Pecio L., Kozachok S., Mirces C., Corciova A., Verestiuc L., Cioanca O., Olezek W., Hancianu M. (2021). Phytochemical profile, antioxidant activity, and cytotoxicity assessment of Tagetes erecta L. flowers. Molecules.

[19] Moliner C., Barros L., Dias M.I., López V., Langa E., Ferreira I.C.F.R., Gómez-Rincón C. (2018). Edible flowers of Tagetes erecta L. as functional ingredients: Phenolic composition, antioxidant and protective effects on Caenorhabditis elegans. Nutrients.

[20] Parejo I., Jáuregui O., Viladomat F., Bastida J., Codina C. (2004). Characterization of acylated flavonoid-O-glycosides and methoxylated flavonoids from Tagetes maxima by LC-ESI-MS/MS. Rapid Commun. Mass. Spectrom..

[21] Luu Q.N., Doorn J.M., Berry M.T., Jiang C., Lin C.F., May P.S. (2011). Preparation and optical properties of silver nanowires and silver-nanowire thin films. J. Colloid. Interf. Sci..

[22] Wang J., An C., Zhang M., Qin C., Ming X., Zhang Q. (2012). Photochemical conversion of AgCl nanocubes to hybrid AgCl-Ag nanoparticles with high activity and long-term stability towads photocatalytic degradation of organic dyes. Can. J. Chem..

[23] Jia C., Yang P., Zhang A. (2014). Glycerol and ethylene glycol co-mediated synthesis of uniform multiple crystalline silver nanowires. Mat. Chem. Phys..

[24] Shankar S., Prasad R.G.S.V., Selvakannan P.R., Jaiswal L., Laxman R.S. (2015). Green synthesis of silver nanoribbons from waste X-ray films using alkaline protease. Mat. Exp..

[25] Hyllested J.A., Espina Palanco M., Hagen N., Mogensen K.B., Kneipp K. (2015). Green preparation and spectroscopic characterization of plasmonic silver nanoparticles using fruits as reducing agents. Beilstein J. Nanotehcnol.

[26] Quinten M. (2001). The color of finely dispersed nanoparticles. Appl. Phys. B.

[27] Shameli K., Bin Ahmad M., Yunus W.Z.W., Ibrahim N.A., Darroudi M. (2010). Synthesis and characterization of silver/talc nanocomposites using the wet chemical reduction method. Int. J. Nanomed..

[28] Venkatesham M., Ayodhya D., Madhusudhan A., Veerabhadram G. (2015). Synthesis of stable silver nanoparticles using gum acacia as reducing and stabilizing agent and study of its microbial properties: A novel green approach. Int. J. Green. Nanotechnol..

[29] Kashyap M., Samadhiya K., Ghosh A., Anand V., Shirage P.M., Bala K. (2019). Screening of microalgae for biosynthesis and optimization of Ag/AgCl nano hybrids having antibacterial effect. RSC Adv..

[30] Alharbi N.S., Alsubhi N.S., Felimban A.I. (2022). Green synthesis of silver nanoparticles using medicina plants: Characterization and application. J. Radiat. Res. Appl. Sci..

[31] Kim K.J., Sung W.S., Suh B.K., Moon S.K., Choi J.S., Kim J.G., Lee D.G. (2009). Antifungal activity and mode of action of silver nanoparticles on Candida Albicans. Biometals.

[32] Zheng K., Setyawati M.I., Leong D.T., Xie J. (2018). Antimicrobial silver nanomaterials. Coord. Chem. Rev..

[33] Li X.Z., Nikaido H., Williams K.E. (1997). Silver resistant mutants of *Eschericha Coli* display active efflux of Ag^+^ and are deficient in Porins. J. Bacteriol..

[34] Levard C., Mitra S., Yang T., Jew A.D., Badireddy A.R., Lowry G.V., Brown G.E. (2013). Effect of chloride on the disolution rate of silver nanoparticles and toxicity to *E. coli*. Environ. Sci. Technol..

[35] Gupta A., Maynes M., Silver S. (1998). Effects of halides on plasmid-mediated silver resistance in *Escherichia coli*. Appl. Environ. Microbiol..

[36] Silver S., Perry R.D., Tynecka Z., Kinscherf T.G. (1982). Mechanisms of bacterial resistances to the toxic heavy metals antimony, arsenic, cadmium, mercury and silver. In Proceedings of Third Tokyo Symposium on Drug Resistance in Bacteria.

[37] Domínguez X. (1973). Métodos de Investigación Fitoquímica.

[38] Ballesteros O.J.V., Perea E.M., Méndez J.J., Arango W.M., Noreña D.A. (2013). Quantification, chemical and biological characterization of the saponosides material from *Sida cordifolia* (escobilla). Rev. Cubana Plant Med..

[39] Scales F.M. (1915). The determination of reducing sugars. J. Bio Chem..

[40] Andrade S., Hypolito R., Ulbrich H.H.G.J., Silva M. (2002). Iron (II) oxide determination in rocks and mineral. Chem. Geo.

[41] Herrera-Gómez A. (2020). Uncertainties in photoemision peak fitting accounting for the covariance with background parameters. J. Vac. Sci. Technol. A.

[42] Vickerman J.C., Gilmore I.S. (2009). Surface Analysis. The Principal Techniques.

[43] Beamson G., Briggs D. (1992). High Resolution XPS of Organic Polymers. The Scienta ESCA300 Databse.

[44] Kahm M., Hasenbrink G., Lichtenberg-Fraté H., Ludwig J., Kschischo M. (2010). grofit: Fitting biological growth curves with R. J. Stat. Softw..

[45] Paul D.J., Laure N.B., Guru S.K., Khan I.A., Ajit S.K., Vishwakarma R.A., Pierre T. (2014). Antiproliferative and antimicrobial activities of alkylbenzoquinone derivatives from Ardisia kivuensis. Pharm. Biol..

